# Overactive Bladder: Pathophysiology, Diagnostics, and Therapies

**DOI:** 10.1155/2011/863504

**Published:** 2012-06-17

**Authors:** John P. F. A. Heesakkers, Francisco Cruz, Yasuhiko Igawa, Ervin Kocjancic

**Affiliations:** ^1^Department of Urology, Radboud University Nijmegen MC, P.O. Box 9101, 6500 HB Nijmegen, The Netherlands; ^2^Department of Urology, University of Porto, 4200-319 Porto, Portugal; ^3^Department of Continence Medicine, The University of Tokyo Graduate School of Medicine, Tokyo 113-8655, Japan; ^4^Department of Urology, College of Medicine, University of Illinois at Chicago, 840 SouthWood Street, Chicago, IL 60612, USA

This special issue on the overactive bladder deals with specific aspects of OAB with respect to pathophysiology, specific patient populations, and treatments. The editorial board selected 8 papers that address these special angles of the OAB spectrum.

In the basic research section, Y. Kubota et al. describe the role of KIT-positive cells in the urinary bladder. KIT staining is used as a marker for interstitial cells. Latest publications suggest that KIT is not only a detection marker of these cells but also may play a crucial role in the control of bladder function.

T. Antunes-Lopes et al. relate about the latest developments of biomarkers like nerve growth factor (NGF) and brain derived neurotrophic factor (BDNF) and how useful they are at this moment to be as biomarkers. It seems that BDNF is the best future candidate for this goal.

With respect to medical treatment, three papers are presented. The first is from A. Athanasopoulos and K. Giannitsas. In an overview about antimuscarinics, they conclude that the differences are small and that individuals respond differently to the various available drugs. A. Matsumori et al. illustrate that it is possible to prescribe other antimuscarinics when the first one fails. They present data about the effect of propiverine in patients who failed other antimuscarinic treatments for OAB. A special group of patients who are treated with OAB is presented by F. van Rey and J. Heesakkers. In a group of MS patients with OAB, the highly beneficial effect of solifenacin is presented based on bladder diaries and patient-reported outcome measures.

Another well-established treatment for OAB is pelvic electrical neuromodulation presented by T. F. Al-Shaiji et al. They review the indications, possible mechanisms of action, surgical aspects and possible complications, and safety issues of this technique.

A completely different type of neurostimulation for OAB is described by F. M. J. Martens et al. They relate about the results of the Finetech-Brindley neurostimulator in patients with a complete spinal cord injury. This difficult and time-consuming surgery has various beneficial effects on as well the urinary and the gastrointestinal tract. Moreover, the sexual functions and the lower limb spasms may change to the good with this exciting technique.

The last contribution in this special issue on OAB is from J. Neuhaus et al. They present about a fairly difficult and special kid on the block: those suffering from bladder pain syndrome or interstitial cystitis. They propose a new diagnostic model that includes extended diagnostics with molecular markers. Differential diagnosis and tailored therapy according to them should be based on three diagnostical columns: clinical diagnostics, histopathology, and molecular diagnostics. The future will tell whether this is way to go in this difficult-to-treat disturbance of the lower urinary tract.

The editorial board of this special issue hopes that these topics give you more insight in the OAB spectrum and that it gives new ideas to implement in basic research as well as clinical practice.

*John P. F. A. Heesakkers* *Francisco Cruz* *Yasuhiko Igawa* *Ervin Kocjancic*

